# Evolutionary Tracks of Chromosomal Diversification in Surgeonfishes (Acanthuridae: *Acanthurus*) Along the World’s Biogeographic Domains

**DOI:** 10.3389/fgene.2021.760244

**Published:** 2021-10-29

**Authors:** Maria Aparecida Fernandes, Marcelo de Bello Cioffi, Luiz Antônio Carlos Bertollo, Gideão Wagner Werneck Félix da Costa, Clóvis Coutinho da Motta-Neto, Amanda Tôrres Borges, Rodrigo Xavier Soares, Allyson Santos de Souza, Krit Pinthong, Weerayuth Supiwong, Alongklod Tanomtong, Wagner Franco Molina

**Affiliations:** ^1^ Department of Cell Biology and Genetics, Biosciences Center, Federal University of Rio Grande do Norte, Natal, Brazil; ^2^ Department of Genetics and Evolution, Federal University of São Carlos, São Carlos, Brazil; ^3^ Department of Fundamental Science, Faculty of Science and Technology, Surindra Rajabhat University, Muang, Thailand; ^4^ Applied Science Program, Faculty of Interdisciplinary Studies, Khon Kaen University, Nong Khai Campus, Nong Khai, Thailand; ^5^ Program of Biology, Faculty of Science, Khon Kaen University, Khon Kaen, Thailand

**Keywords:** marine fish, comparative cytogenetic, hisDNA, oceanic barrier, multigenic family

## Abstract

Fishes of the genus *Acanthurus* (Acanthuridae) are strongly related to reef environments, in a broad biogeographic context worldwide. Although their biological aspects are well known, cytogenetic information related to this genus remains incipient. In this study, *Acanthurus* species from populations inhabiting coastal regions of the Southwest Atlantic (SWA), South Atlantic oceanic islands (Fernando de Noronha Archipelago and Trindade Island), Greater Caribbean (GC), and Indo-Pacific Ocean (the center of the origin of the group) were analyzed to investigate their evolutionary differentiation. For this purpose, we employed conventional cytogenetic procedures and fluorescence *in situ* hybridization of 18S rDNA, 5S rDNA, and H3 and H2B-H2A histone sequences. The Atlantic species (*A. coeruleus, A. chirurgus*, and *A. bahianus*) did not show variations among them, despite their vast continental and insular distribution. In contrast, *A. coeruleus* from SWA and GC diverged from each other in the number of 18S rDNA sites, a condition likely associated with the barrier created by the outflows of the Amazonas/Orinoco rivers. The geminate species *A. tractus* had a cytogenetic profile similar to that of *A. bahianus*. However, the chromosomal macrostructures and the distribution of rDNA and hisDNA sequences revealed moderate to higher rates of diversification when *Acanthurus* species from recently colonized areas (Atlantic Ocean) were compared to *A. triostegus*, a representative species from the Indian Ocean. Our cytogenetic data covered all *Acanthurus* species from the Western Atlantic, tracked phylogenetic diversification throughout the dispersive process of the genus, and highlighted the probable diversifying role of ocean barriers in this process.

## Introduction

Acanthuridae (surgeonfishes, tangs, and unicornfishes) represents a charismatic group of primarily large-bodied herbivorous fish species, which play an important ecological role in benthic communities and the resilience of coral environments in all tropical and subtropical seas (Randall, 2001; Green and Bellwood, 2009; Russ et al., 2018). The common name “surgeonfish” refers to the peculiar scalpel-like modified scales on both sides of the caudal peduncle that is used in inter- and intraspecific aggressive interactions (Randall, 2001). Due to their attractive colors and shapes, surgeonfishes dominate aquarium trade in several areas (Sadovy and Vincent, 2002; Papavlasopoulou et al., 2014).

The family comprises six genera and 85 species (Fricke et al., 2021). *Acanthurus* is the most diverse clade with 40 species, of which 90% are endemic to the Indo-Pacific, the origin and dispersion center of the group (Randall, 2001). Despite the growing set of genetic data on Acanthuridae, spanning the nuclear (Bernardi et al., 2018) and the mitochondrial (Ludt et al., 2020) genomes, the cytogenetic features of Acanthuridae are still largely incipient (<10% of species) (Arai and Inoue, 1976; Affonso et al., 2014; Fernandes et al., 2015).

Four species of *Acanthurus*: *A. coeruleus* (blue tang surgeonfish), *A. bahianus* (barber surgeonfish), *A. chirurgus* (doctorfish), and *A. tractus* (ocean surgeonfish), are found in the Western Atlantic, with an extensive distribution from the Caribbean to southern Brazil, including the island regions of Fernando de Noronha and Trindade and Martim Vaz (Rocha et al., 2002). In addition, the wide distribution of *Acanthurus* around the world offers a suitable model for investigating chromosomal specificities fixed among populations in the marine environment. Several species of *Acanthurus* are found in the Pacific and Indo-Pacific oceans, the center of origin of the genus, among which *A. triostegus* (convict surgeonfish) is one most representative species. *Acanthurus triostegus* has a remarkable ability to extend its larval stage by slowing metamorphosis (McCormick, 1999), with populations between the Indian and Pacific oceans exhibiting genetic structure (Grulois et al., 2020). Currently, the karyotype of this species is only known in representatives of the Pacific regions (Arai and Inoue, 1976; Ojima and Yamamoto, 1990); comparisons with samples from the Indian Ocean will be useful for estimating evolutionary divergences across the Indo-Pacific region. Concerning the Western Atlantic region, the freshwater outflows from the Amazonas (Brazil) and Orinoco (Venezuela) rivers delimit the Brazilian and the Greater Caribbean (GC) biogeographic provinces (Floeter et al., 2008). Although this barrier interferes with the genetic structure of some *Acanthurus* species (Rocha et al., 2002; Rocha 2003), it is not yet known whether the barrier contributes to karyotype differentiation between divided populations.

Due to the high evolutionary dynamic of multigene families, such as the ribosomal DNA genes (Gornung, 2013), with a vital role in protein synthesis, and of histones (H1, H2B-H2A, H3, and H4), acting in the structural organization of chromatin and regulation of gene expression in eukaryotes (Chioda et al., 2002), they have been largely used in evolutionary and population approaches (e.g., Motta-Neto et al., 2012; Amorim et al., 2017). The preferential participation of concerted evolution in the ribosomal DNA (Gonzalez and Sylvester, 2001) or by birth-and-death evolution in histone genes (Rooney and Ward, 2005) promotes differential arrays that can be followed in the genome of the species and populations (Lima-Filho et al., 2012; Costa et al., 2016).

Chromosomal variations result in the amplification of adaptive aspects of species (Kess et al., 2020). Additionally, micro or macrostructural cytogenetic patterns can be correlated with the species distribution, and action of biogeographic barriers (Motta-Neto et al., 2019), and thus associated to the diversification of Acanthuridae in the marine environment. Although mapping of repetitive DNA sequences has produced effective cytogenetic markers for detecting cryptic evolutionary diversification among Atlantic reef fishes (Costa et al., 2014; Amorim et al., 2016; Nirchio et al., 2017), little is known about the chromosomal organization in Acanthuridae. In this study, the chromosomal distribution of rDNA and histone genes among five species of *Acanthurus* from the Southwest Atlantic (SWA), Greater Caribbean (GC), and Indian Ocean (IO) were utilized to track their karyotype evolution and assess population stratifications. The results reveal a panel of increasing karyotype diversification associated with the historical biogeography of *Acanthurus* and present evidence of intraspecific variability between Atlantic areas.

## Materials and Methods

### Sampling and Chromosome Preparation

Individuals of *A. coeruleus*, *A. bahianus*, *A. tractus*, and A*. chirurgus* were collected from different regions of the Western Atlantic, Brazilian Northeast coast (Rio Grande do Norte State), insular Atlantic regions (Fernando de Noronha archipelago and Trindade Island), and Florida Keys, an island archipelago in southern Florida (United States), belonging to GC province. *Acanthurus triostegus* individuals were obtained from the Andaman Sea in the Indian Ocean (Table 1).

**TABLE 1:** Data of the species and populations of *Acanthurus* (Acanthuridae) used in cytogenetic analyses.

Species	Oceanic regions					N
	Southwest Atlantic (SWA)	Oceanic Atlantic islands		Greater Caribbean (GC)	Indian ocean	
	Brazilian coast (Rio Grande do Norte State)	Fernando de Noronha Archipelago	Trindade Island	Florida keys (Florida State, United States)	Andaman Sea	
*A. coeruleus*	31	01	—	08	—	40
*A. bahianus*	15	—	05	—	—	20
*A. tractus*	—	—	—	02	—	02
*A. chirurgus*	11	04	—	01	—	16
*A. triostegus*	—	—	—	—	03	03

All field and laboratory protocols used in this study, including specimen sampling, were approved by the Ethics Committee on the Use of Animals at the Federal University of Rio Grande do Norte (Proc.#044-15). Sample collections were authorized by the Chico Mendes Institute for Biodiversity Conservation (ICMBio), System of Authorization, and Information on Biodiversity (SISBIO-Licenses No. 19135-1, 131360-1 and 27,027-2).

The specimens were subjected to *in vivo* mitotic stimulation for 24 h using an attenuated antigen complex (Molina et al., 2010). The chromosome preparations were performed in short-term culture according to Gold et al. (1990). Silver-stained nucleolus organizer regions (Ag-NORs) and heterochromatic regions were visualized following the protocols described by Howell and Black (1980) and Sumner (1972), respectively.

### Barcoding of Cryptic Species

Because of the cryptic identification of *A. bahianus* and the newly resurrected *A. tractus* species (Bernal and Rocha, 2011) and their sympatric occurrence in GC (Castellanos-Gell et al., 2012), genetic analyses using sequences of the cytochrome oxidase I (COI) were performed to confirm their taxonomic status. For this purpose, fragments of fins were removed, preserved in 95% ethanol, and stored at 4 °C. The total DNA of each specimen was extracted (Sambrook et al., 1989) and amplified by polymerase chain reaction (PCR) using primers for the COI gene. The PCR reactions consisted of 1 μL of total DNA, 0.5 U Taq polymerase, 0.4 μL of 50 mM MgCl_2_, 1 μL of 10 x buffer, 0.5 μL 10 mM dNTP, 0.3 μL of each primer (10 μM) (L1987 and H2609) (Palumbi, 1991), and ultrapure water until the final volume of 25 μL. The amplification reactions were performed with an initial denaturation cycle at 95°C for 5 min; followed by 30 cycles at 94°C for 30 s, 49°C for 30 s, 72°C for 55 s, and a final extension at 72°C for 5 min. The PCR products were purified using the enzyme ExoSAP-IT (Applied Biosystems, Waltham, Massachusetts, EUA) and sequenced by ACTGene Ltd. The COI gene sequences of the individuals were compared to GenBank (www.ncbi.nlm.nih.gov/genbank/) and BOLD (www.boldsystems.org) databases employing BOLD identification tools and Blastn Search Tool, respectively, with those of *A. tractus*, confirming its taxonomic status.

### Probes Preparation

The 5S rDNA (200 bp) and 18S rDNA (1,400 bp) probes were obtained by PCR from the nuclear DNA of *A. coeruleus* individuals from Northeast Brazilian coast using primers A 5′-TAC GCC CGA TCT CGT CCG ATC-3′ and B 5′ -CAG GCT GGT ATG GCC GTA AGC-3' (Pendás et al., 1994) and NS1 5′-GTA GTC ATA TGC TTG TCT C-3′ and NS8 5′-TCC GCA GGT TCA CCT ACG GA-3′ (White et al., 1990), respectively. The 5S rDNA and 18S rDNA probes were labeled with biotin-14-dATP and digoxigenin-11-dUTP, respectively, using nick translation according to the manufacturer’s recommendations (Roche, Mannheim, Germany). Meanwhile, primers H2BAD 5′-CCC -CCC GAG ATG TGA TGG TAG A-3 ′ and H2BAR 5′-AGT ACA GCC TGG ATG TTT GGT AA-3′ were used to amplify the H2B-H2A sequences and primers H3D 5′-ATG GCT CGT ACC AAG CAG ACV GC-3′ and H3R 5′-ATA TCC TTR GGC ATR ATR GTG AC-3′ to amplify H3 sequences. Both primer sets were designed using the gene sequences of *Mytilus edulis* (Albig et al., 2003), and the genes were amplified according to Giribert and Distel (2003). Biotin-14-dATP and digoxigenin-11-dUTP were used to label H2B-H2A hisDNA and H3 hisDNA, respectively, using nick translation according to the manufacturer’s recommendations (Roche, Mannheim, Germany).

### Fluorescence *in situ* Hybridization (FISH)

FISH experiments were performed according to the protocols described by Pinkel et al. (1986). Mitotic chromosomes were treated with RNAse (20 μg/ml in 2 x SSC) at 37°C for 1 h and then with pepsin (0.005% in 10 mM HCl) at 37°C for 10 min, fixed with 1% formaldehyde for 10 min, and dehydrated using a series of alcohol solutions (70,85, and 100%) for 5 min. The chromosomal preparations were incubated in 70% formamide/2 × SSC at 72°C for 5 min. The hybridization solution ((50% formamide, 2 x SSC, and 10% dextran sulfate) and the denatured probe (5 ng/μL), with a final volume of 30 μL, were deposited on the slides, and hybridization was performed for 16 h at 37°C. Post-hybridization washes were performed using 15% formamide/0.2 × SSC at 42°C for 20 min, followed by washes using 0.1 × SSC at 60°C for 15 min and 0.5%/4 × SSC Tween 20 for 5 min at 25°C. Hybridization signals were detected using rhodamine-conjugated anti-digoxigenin for 18S rDNA and H3 hisDNA probes and FITC-conjugated streptavidin (Vector, Burlingame, CA, United States) for 5S rDNA and H2B-H2A hisDNA. Chromosomes were counterstained with Vectashield antifade with DAPI (4′,6-diamidine-2-phenylindole dihydrochloride) (1.5 μg/ml; Vector Laboratories Burlingame, CA, United States).

### Microscopy and Image Analyses

Chromosomal images were obtained using an Olympus BX51 epifluorescence photomicroscope (Olympus, Tokyo, Japan) coupled to an Olympus DP73 digital capture system using the cellSens software (Olympus, Tokyo, Japan). Chromosomes were classified as metacentric (m), submetacentric (sm), subtelocentric (st), and acrocentric 1) according to their arms ratio (Levan et al., 1964). The fundamental number (FN) was established considering the occurrence of two arms on the meta-, submeta-, and subtelocentric chromosomes, and only one on the acrocentric chromosomes. Karyotypes were organized according to the decreasing order of size of the chromosomes within each of their respective morphological classes. Ideograms ​​representing chromosomes and repetitive DNA class arrays were prepared using the Photoshop CS6 software.

## Results

**FIGURE 1:**
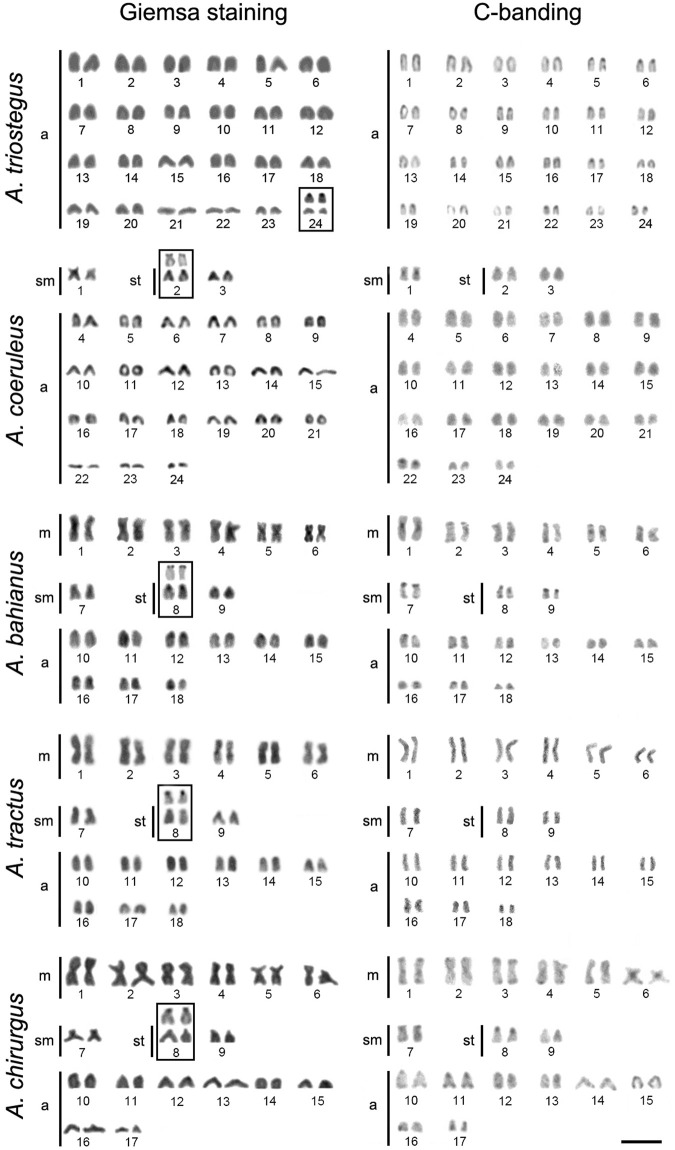
Karyotypes of *Acanthurus triostegus*, *Acanthurus coeruleus*, *Acanthurus tractus*, *Acanthurus bahianus*, and *Acanthurus chirurgus* after Giemsa-staining and C-banding. Boxes highlight the chromosome pair with Ag-NORs (silver stained NORs). m–metacentric, sm–submetacentric, and a–acrocentric chromosomes. Scale bar = 5 μm.

*Acanthurus triostegus* from the Andaman Sea, analyzed for the first time has 2n = 48 acrocentric chromosomes (FN = 48), with Ag-NOR sites located in the short arm of pair 24. Meanwhile, *A. coeruleus* (2n = 48; 2 s + 4th + 42a; FN = 54), *A. tractus* (2n = 36; 12 m + 2 s + 4th + 18a; FN = 54), *A. bahianus* (2n = 36; 12 m + 2 s + 4th + 18a; FN = 54), and *A. chirurgus* (2n = 34; 12 m + 2 s + 4th + 16a; FN = 52) presented karyotype patterns similar to those previously reported (Affonso et al., 2014; Fernandes et al., 2015) (Figure 1). Different populations of *A. coeruleus*, *A. chirurgus*, and *A. bahianus* (Table 1) displayed similar karyotype structures when compared to each other.

**FIGURE 2:**
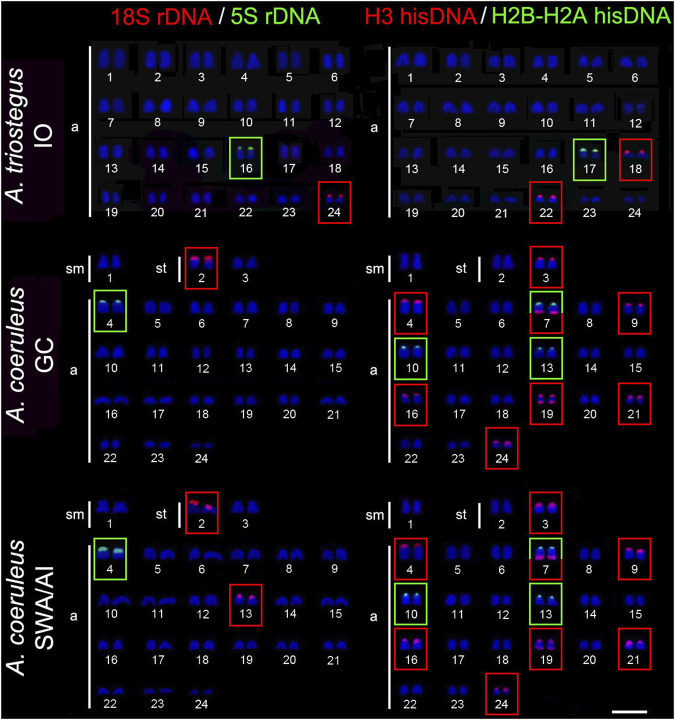
Fluorescence *in situ* hybridization signals indicating the distribution of the 18S rDNA (red signals), 5S rDNA (green signals), H3 hisDNA (red signals) and H2B-H2A hisDNA (green signals) sites in chromosomes of *Acanthurus triostegus* and *Acanthurus coeruleus*. Scale bar = 5 μm. IO, Indian Ocean; SWA, Southwest Atlantic; AI, South Atlantic Islands; GC, Greater Caribbean.

**FIGURE 3:**
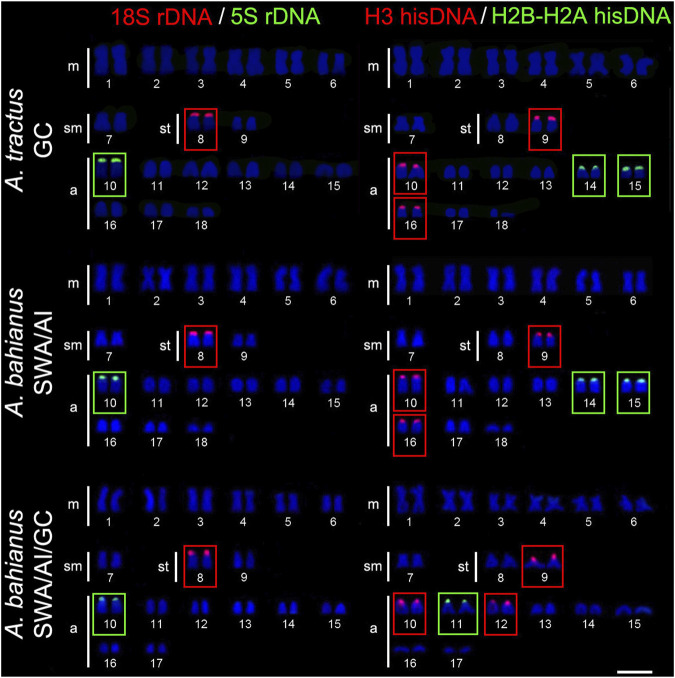
Fluorescence *in situ* hybridization signals showing the distribution of the 18S rDNA (red signals), 5S rDNA (green signals), H3 hisDNA (red signals) and H2B-H2A hisDNA (green signals) sites in chromosomes of *Acanthurus tractus*, *Acanthurus bahianus*, and *Acanthurus chirurgus*. Scale bar = 5 μm. SWA, Southwest Atlantic; AI, South Atlantic Islands; GC, Greater Caribbean.

The 18S rDNA sites occur exclusively in the short arm of pair 24 in *A. triostegus*, meanwhile, the sites are located in the short arms of the largest subtelocentric pair (pair 8), without inter-population variability, in *A. tractus*, *A. bahianus*, and *A. chirurgus*. In contrast, the number of 18S rDNA sites in *A. coeruleus* differs between individuals from the Brazilian coast (two loci in the short arms of pairs 2 and 13) –and from the Florida Keys (only one locus in the short arms of pair 2) (Figures 2, 3).

The 5S rDNA sites are located in the short arms of pair 16 in *A. triostegus*. In the Atlantic species, *A. coeruleus*, *A. tractus*, *A. bahianus*, and *A. chirurgus*, the sites occur in the short arms of the largest acrocentric pair: pairs 4, 10, and 10 in individuals from Florida Keys (GC), Brazilian coast (SWA), and Atlantic oceanic islands, respectively (Figures 2, 3).

In all species, the H2B-H2A hisDNA sites are located exclusively in the short arms of the chromosomes but with numerous divergences among them: only one locus in *A. triostegus* (pair 17), only one locus also in *A. chirurgus* (pair 11), two loci in *A. tractus* and *A. bahianus* (pairs 14 and 15), and three loci in *A. coeruleus* (pairs 7, 10 and 13), highlighting a syntenic array with the H3 hisDNA in pair 7 (Figures 2, 3). In turn, the H3 hisDNA showed notable variations in the number of loci and distribution among species. In *A. triostegus*, two loci were located in the short arms of pairs 18 and 22 (Figure 2). In *A. coeruleus* nine loci, the largest number among species, are distributed in the short arms of pairs 3, 4 (co-located with a 5S rDNA site), 16, 21, and 24, and in the terminal regions of the long arms of pairs 7 and 19, in this latter with a bitelomeric arrangement (Figure 2). Meanwhile, *A. tractus* and *A. bahianus* presented three loci: in the short arms of pairs 9, 10 (co-localized with 5S rDNA site), and in the short arms of pair 16. *Acanthurus chirurgus* also showed three loci: in the short arms of pairs 9, 10 (co-localized with 5S rDNA site) and in pair 12 (Figure 3).

## Discussion

The historical biogeography and current geographic context of *Acanthurus* species offer useful conditions for estimating the putative effects promoted by barriers and large oceanic spaces. In this context, our present data demonstrated cytogenetic variations and large-scale karyoevolutionary changes among populations and species, highlighting varied levels of divergence.

### Karyotype Diversification in Western Atlantic Species of *Acanthurus*

Geographic barriers and ecological limitations can promote genetic structuring and endemism in coastal areas and oceanic islands of the Western Atlantic (Pinheiro et al., 2018). Karyotype divergences have been identified among several reef fish species divided by the Amazonas/Orinoco river plume (Nirchio et al., 2008; Rocha and Molina, 2008; Motta-Neto et al., 2019), probably promoted by divergent evolutionary forces under gene flow limitation.

*Acanthurus* species currently inhabiting the Western Atlantic are derived from two lineages that reached this oceanic region by different routes of colonization. Inferences based on mitochondrial and nuclear genetic sequences and fossil information suggest that one of them, reaching the region through the Tethys seaway, gave rise to *A. bahianus*, *A. tractus*, and *A. chirurgus*, at around 17.1 Mya (14.5–24.8 95% highest posterior density, HPD). The other one, coming through the Isthmus of Panama, colonized more recently the Western Atlantic, at around 13.1 Mya (7.6–18.8 HPD) (Siqueira et al., 2019). *Acanthurus coeruleus* (2n = 48), whose Atlantic colonization is derived from the Pacific lineage, has the largest number of acrocentric chromosomes, sharing the greatest karyotype similarity with *A. triostegus* (2n = 48a), a basal *Acanthurus* species from the Indo-Pacific Ocean (Sorenson et al., 2013). In general, conspicuous series of sequential rearrangements mainly derived from pericentric inversions, centric and *in tandem* fusions, promoted the karyotype diversification in Atlantic *Acanthurus* (Affonso et al., 2014).

**FIGURE 4:**
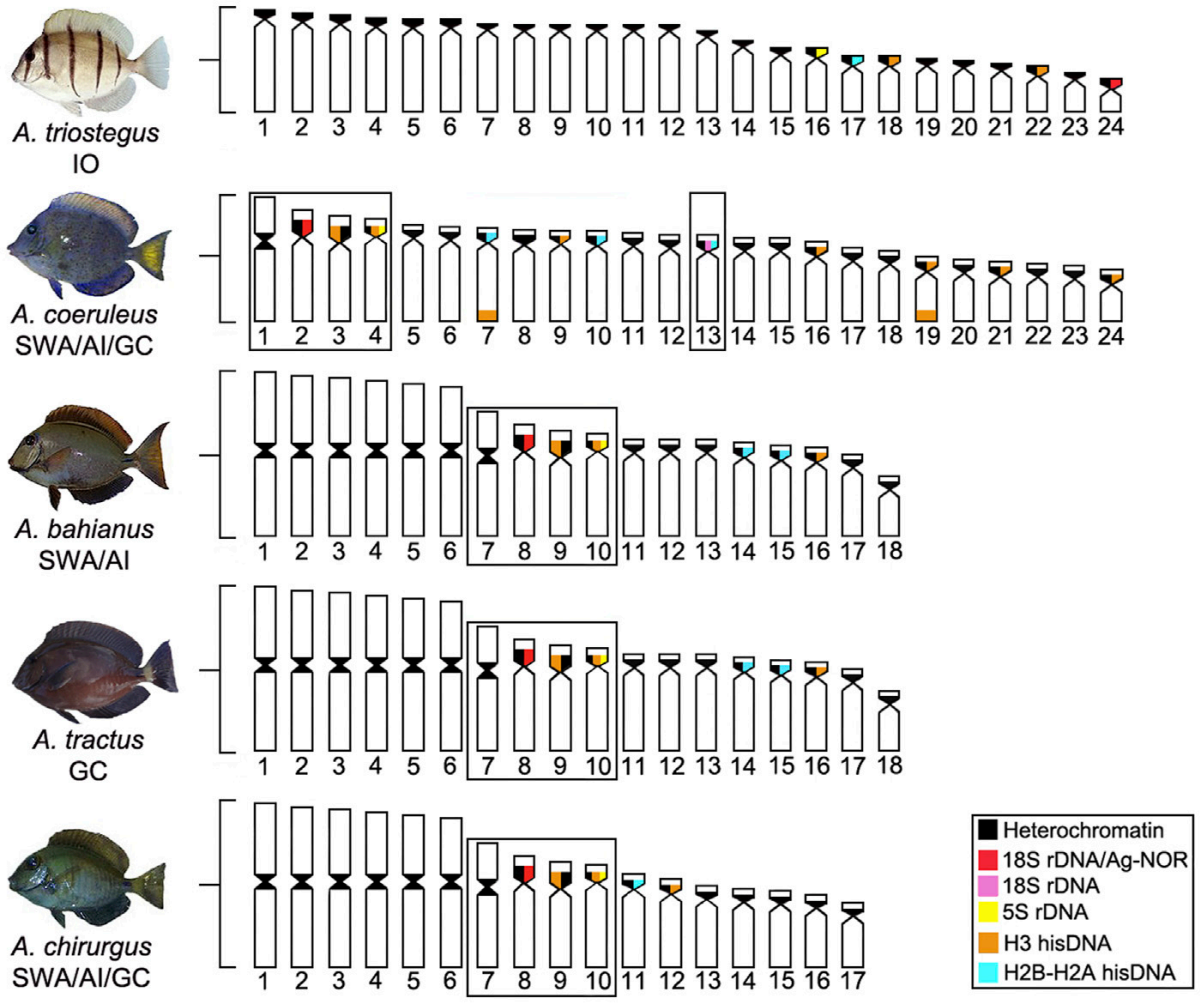
Ideogram showing the organization of different classes of repetitive DNAs (rDNA and hisDNA) in five species and populations of *Acanthurus*. The boxes highlight a set of two-armed chromosomes shared by all Atlantic species. The dotted box highlights a chromosome pair bearing an extra 18S rDNA site in Southwest Atlantic individuals. GC, Greater Caribbean; SWA, Southwest Atlantic; IO, Indian Ocean.

Given the current known biogeographic history, the evolutionary origin (homologous or homoplasic) of the set of three pairs of two-armed chromosomes in all the Atlantic *Acanthurus* species (Figure 4) deserves further investigation, given that reconstructed phylogenetic relationships based in molecular evidences indicate that *A. coeruleus* is phylogenetically distant from *A. bahianus*, *A. tractus*, and *A. chirurgus* (Sorenson et al., 2013). However, the common origin of this set of chromosomes by pericentric inversions in *A. tractus*, *A. bahianus*, and *A. chirurgus*, as well as of the identical set of six large metacentric pairs by Robertsonian fusions and with a similar distribution of repetitive sequences (Affonso et al., 2014; Fernandes et al., 2015; present work), indicate a synapomorphic condition fixed among these Western Atlantic species.

The evolutionary split between *A. tractus* and *A. bahianus* was preliminarily estimated at around one Mya. (Castellanos-Gell et al., 2012). Far from the origin of the Amazonas-Orinoco freshwater outflow barrier (around 10 Mya; Lovejoy et al., 1998), this recent event was not enough to fixate differential chromosomal characteristics between these two species. As these geminate species share similar karyotype patterns and have secondary contacts due to punctuated invasions of *A. bahianus* from the north to the south Atlantic regions in more recent times (Castellanos-Gell et al., 2012), they are very suitable targets for further evolutionary investigations. In fact, the sympatry between these geminate species is a scenario propitious to introgressive hybridization, a recurrent condition in *Acanthurus* species (Marie et al., 2007; DiBattista et al., 2016) and with evolutionarily consequences to Caribbean populations. On the other hand, the karyotype of *A. chirurgus* is significantly differentiated by its smaller diploid number (2n = 34) than that of *A. bahianus* and *A. tractus* (2n = 36). This autapomorphic condition is likely derived from *in tandem* fusion between a small acrocentric and the larger submetacentric chromosome pairs (Affonso et al., 2014).

Successive chromosomal differentiation in number and structure has occurred throughout the evolution of the Atlantic *Acanthurus* karyotypes; however, microstructural changes involving the repetitive fraction of the genome show diverse patterns. Repetitive sequences are important components of genomic differentiation and evolutionary processes (López-Flores and Garrido-Ramos, 2012; Biscotti et al., 2015). Eventually, these redundant sequences are also efficient as population (Lima-Filho et al., 2012) or cytotaxonomic markers (Amorim et al., 2016), even in groups with marked chromosomal conservatism (Motta-Neto et al., 2011a; Calado et al., 2013).

Indeed, while the distribution of the rDNA sequences presents a more regular distribution pattern (Fernandes et al., 2015), our present data show that histone sequences have a more dynamic diversification among species. The histone multigene family is known to play a fundamental role in the structural organization of chromatin in eukaryotes, as well as in the regulation of gene expression (Chioda et al., 2002), showing a considerable level of organization on the chromosomes of various fish groups (Hashimoto et al., 2011; Costa et al., 2016; Borges et al., 2019). Within *Acanthurus*, the Atlantic species (*A. tractus*, *A. bahianus*, *A. chirurgus*, and mainly *A. coeruleus*) show a more variable chromosomal distribution for the H2B/H2A and H3 histones than the Indo-Pacific lineage (*A. triostegus*). In fact, among the Atlantic species, such histone sites are clustered in multiple chromosome pairs, including bitelomeric arrays in some chromosome pairs of *A. coeruleus*.

Except for a few H3 sites occupying telomeric positions in *A. coeruleus*, hisDNA sites are mainly present in centromeric heterochromatic regions. Similar patterns in other fish groups reinforce their functional co-localization with complex sets of repetitive DNAs (Hashimoto et al., 2011; Lima-Filho et al., 2012), including transposable elements (Roehrdanz et al., 2010; Costa et al., 2014, 2016). Based on provisional chromosome arrangements, our FISH signals also indicate the overlapping of H2B/H2A and H3 histones sequences with each other and with rDNA sequences, both 18S and 5S rDNA, in some heterochromatic regions. The association of distinct DNA classes highlights multiple evolutionary processes that model the repetitive DNA fraction in *Acanthurus* species. Additionally, H3 hisDNA showed more abundant sites than H2B-H2A. Despite its uncertain cause, this frequency has also been observed in other marine species, such as *Rachycentron canadum* (Rachycentridae) (Costa et al., 2014), *Ocyurus chrysurus* (Lutjanidae) (Costa et al., 2016), and *Centropomus* species (Centropomidae) (Borges et al., 2019). The number of H2B-H2A and H3 sites declined with respect to the evolutionary divergence of the Atlantic species: *A. coeruleus* with three and eight sites; *A. tractus* and *A. bahianus* with two and three sites; and *A. chirurgus* with one and three sites, respectively. Meanwhile, *A. triostegus* presents the lowest frequency: one and 2 sites. The distribution of hisDNA in these species indicates an evolution by a stochastic birth-and-death process (Novozhilov et al., 2006), with the reduction of sites along with their diversification steps.

### Karyoevolution of the Genus *Acanthurus* in the Biogeographic Context

The cytogenetic profiles of *Acanthurus* species reveal evolutionary steps of chromosomal organization at the macro and microstructural levels, which are supported by historical biogeographic events and phylogenetic relationships. The occurrence of exclusive 2n = 48 acrocentric chromosomes in *A. triostegus*, a basal species with divergence contemporary to the genus *Acanthurus* (Sorenson et al., 2013), to the ancient genus *Prionurus* (*P. microlepidotus*; Arai and Inoue, 1976), and the paraphyletic *Ctenochaetus* (Ojima and Yamamoto, 1990), consolidates this karyotype as baseline for Acanthuridae in a most parsimonious manner. In addition, *A. triostegus* shares other symplesiomorphic traits with several Percomorpha groups, such as unique Ag-NORs/18S rDNA sites and heterochromatin restricted to centromeric regions (Galetti et al., 2000).

In addition to biological factors interfering with the gene flow of marine fish populations (McCormick, 1999; Otwoma et al., 2018; Grulois et al., 2020), stochastic physical events (tectonic processes, glaciations, and opening or closure of oceanic barriers) have modeled the complex biogeography of Acanthuridae (Siqueira et al., 2019) and its karyotype diversification. Cytogenetic comparisons in Atlantic *Acanthurus* highlight conspicuous synapomorphies regarding the karyotype structure and the organization of repetitive DNA classes, concerning the most basal pattern represented by the *A. triostegus* lineage.

**FIGURE 5:**
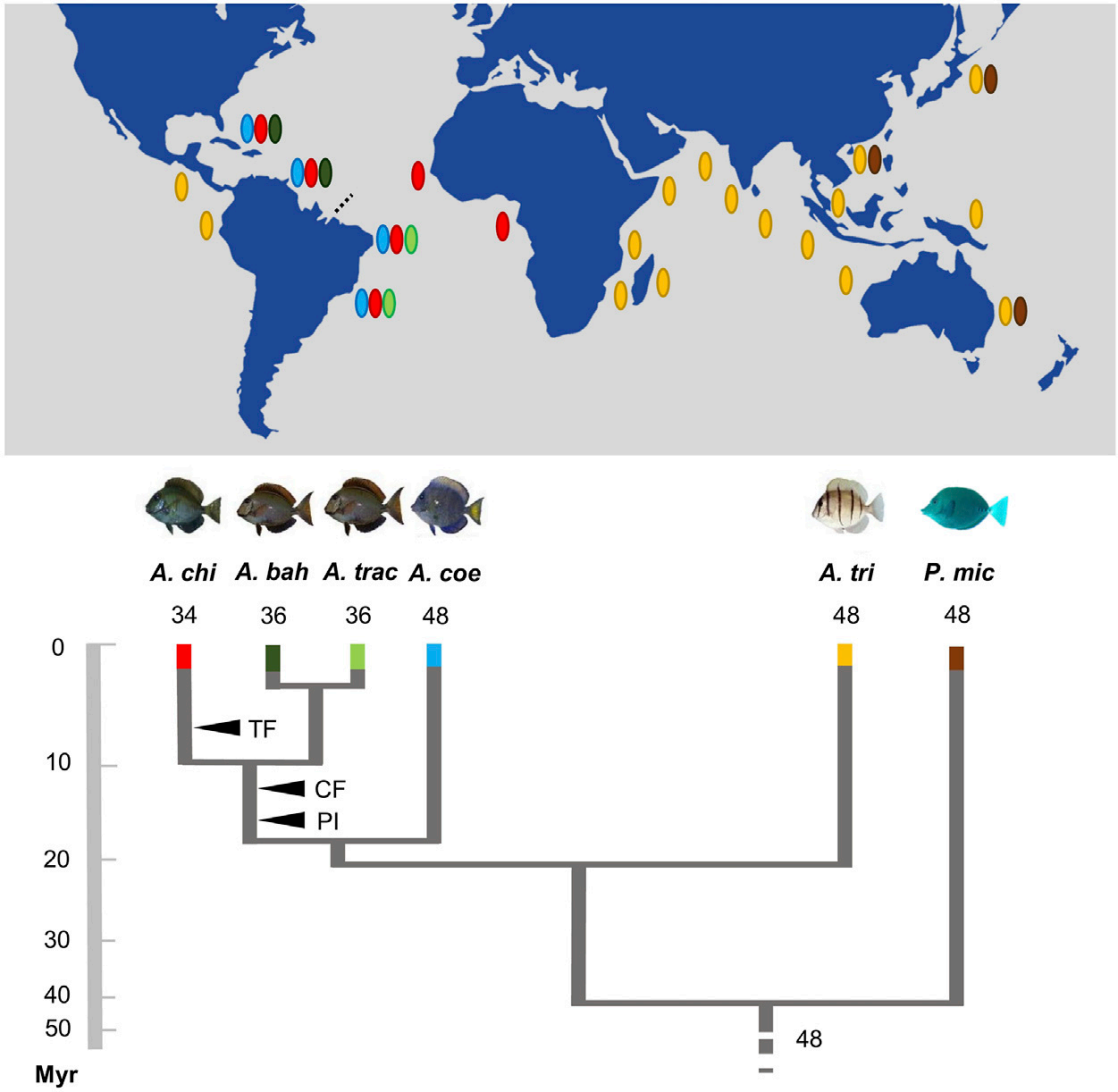
Karyotype diversification, phylogenetic relationships, and schematic geographic distribution of the *Acanthurus* species analyzed. The black arrowheads indicate shared and exclusive chromosomal rearrangements in the Atlantic species (CF: six centric fusions events; PI: three pericentric inversions events; TF: in tandem fusion) regarding their phylogenetic diversification (adapted from Bernal and Rocha, 2011; Sorenson et al., 2013). The dotted line highlights the Amazonas River plume. The names of the species are identified by abbreviations—*Acanthurus chirurgus (A. chi); Acanthurus bahianus (A. bah); Acanthurus tractus (A. tra); Acanthurus coeruleus (A. coe); Acanthurus triostegus (A. tri); Prionurus microlepidotus (P. mic)*. It shows an inverted geographic distribution to the species *Acanthurus tractus* and *Acanthurus bahianus* analyzed.

The dispersive potential of *A. triostegus* (Fisher and Hogan, 2007; Liggins et al., 2016; Otwoma et al., 2018) favor gene flow in the marine environment (Rocha, 2003). These characteristics are possible explanations for the conservative basal karyotype of this species and the karyotype homogeneity between the Indian and Pacific populations. In contrast, *A. coeruleus*, *A. chirurgus*, and *A. tractus*/*A. bahianus*, with recent divergence in the Atlantic, and under different evolutionary and ecological changes, such as allopatry, population fragmentation, and niche displacement (Siqueira et al., 2019), show a more evident chromosomal diversification. Indeed, the divergence between the *A. coeruleus* lineage and the clade composed of *A. tractus*/*A. bahianus* and *A. chirurgus* (19 Mya), and those between the two last clades (10 Mya) (Figure 5), coincides with global changes in ocean dynamics (tectonic events) that affected the levels of richness and endemism of reef fish (Floeter et al., 2008). In the Atlantic Ocean, the isolation of coastal habitats during glaciations, which promoted recurrent variations at sea level, has influenced the patterns of genetic structuring (Souza et al., 2015) and, in more recent times, it may have contributed to a higher rate of chromosome evolution in reef fish.

In general, acanthurids have a very low population structure, even at great distances, such as the 3,540 km between the Brazilian region and Santa Helena Island (Rocha et al., 2002). It is believed that the high potential for dispersion (Lessios and Robertson, 2006) and colonization events of *Acanthurus* (Otwoma et al., 2018) promote genetic homogeneity and limited evolutionary dynamism among species karyotypes. In this context, the karyotype characteristics of Atlantic island populations are only now being clarified. Cytogenetic comparisons between *A. chirurgus* from the Fernando de Noronha Archipelago (FNA) and *A. bahianus* from Trindade Island, and among *A. coeruleus* from FNA, the Brazilian coast, and GC indicated that, despite the great oceanic distances and geographic isolation, there was no noticeable variation in their karyotypes.

In contrast, a certain level of variation occurs between specimens of *A. coeruleus* from GC and the SWA, in which there are two additional 18S rDNA sites in the latter. These additional sites correspond to non-active NORs (negative Ag-NORs) and a cytogeographic characteristic of this population. Significantly, this rDNA marker complements other phylogeographic pieces of evidence indicating population stratification between these areas (Rocha et al., 2002), as a consequence of the outflow barrier from the Amazon and Orinoco rivers. On the other hand, the geminate species, *A. bahianus* and *A. tractus*, resulting from their high sensibility to ecological effects of the Amazonas-Orinoco outows (Rocha et al., 2002), share a common cytogenetic pattern. Similar to some other reef fishes (Getlekha et al., 2018), these commonalities are derived from the recent evolutionary diversification of these groups, which impairs the fixation of chromosomal rearrangements. The sympatric occurrence of *A. bahianus* and *A. tractus* in some areas of the GC (Castellanos-Gell et al., 2012) opens a particular condition for investigating reproductive isolation in the absence of conspicuous karyotype diversification.

It is noteworthy that karyotype variability and diversification between populations and species, such as Lutjanidae (Nirchio et al., 2008; Rocha and Molina, 2008; Costa et al., 2016), Haemulidae (Motta-Neto et al., 2011b), and Grammatidae (Molina et al., 2012), have also been observed in Atlantic regions. These data increase the evidence of the primary interference of the Amazonas/Orinoco River plume in the karyotype differentiation of Atlantic reef fishes. Overall, our data highlight the use of integrating chromosomal patterns, including microstructural characters, phylogenetics, and historical biogeography, in elucidating the karyotype evolution of marine fishes, connectivity among biogeographic provinces and on the estimation of chromosomal divergences among marine populations.

## Data Availability

The original contributions presented in the study are included in the article/supplementary material, further inquiries can be directed to the corresponding author.
