# Corrigendum: Evolutionary tracks of chromosomal diversification in surgeonfishes (Acanthuridae: *Acanthurus*) along the world’s biogeographic domains

**DOI:** 10.3389/fgene.2024.1390546

**Published:** 2024-03-11

**Authors:** Maria Aparecida Fernandes, Marcelo de Bello Cioffi, Luiz Antônio Carlos Bertollo, Gideão Wagner Werneck Félix da Costa, Clóvis Coutinho da Motta-Neto, Amanda Tôrres Borges, Rodrigo Xavier Soares, Allyson Santos de Souza, Krit Pinthong, Weerayuth Supiwong, Alongklod Tanomtong, Wagner Franco Molina

**Affiliations:** ^1^ Department of Cell Biology and Genetics, Biosciences Center, Federal University of Rio Grande do Norte, Natal, Brazil; ^2^ Department of Genetics and Evolution, Federal University of São Carlos, São Carlos, Brazil; ^3^ Department of Fundamental Science, Faculty of Science and Technology, Surindra Rajabhat University, Muang, Thailand; ^4^ Applied Science Program, Faculty of Interdisciplinary Studies, Khon Kaen University, Nong Khai Campus, Nong Khai, Thailand; ^5^ Program of Biology, Faculty of Science, Khon Kaen University, Khon Kaen, Thailand

**Keywords:** marine fish, comparative cytogenetic, hisDNA, oceanic barrier, multigenic family

In the published article, there was an error in Figure 5 as published. It shows an inverted geographic distribution to the species *Acanthurus tractus* and *Acanthurus bahianus* analyzed. The corrected Figure 5 and its caption appear below.

**FIGURE 5:**
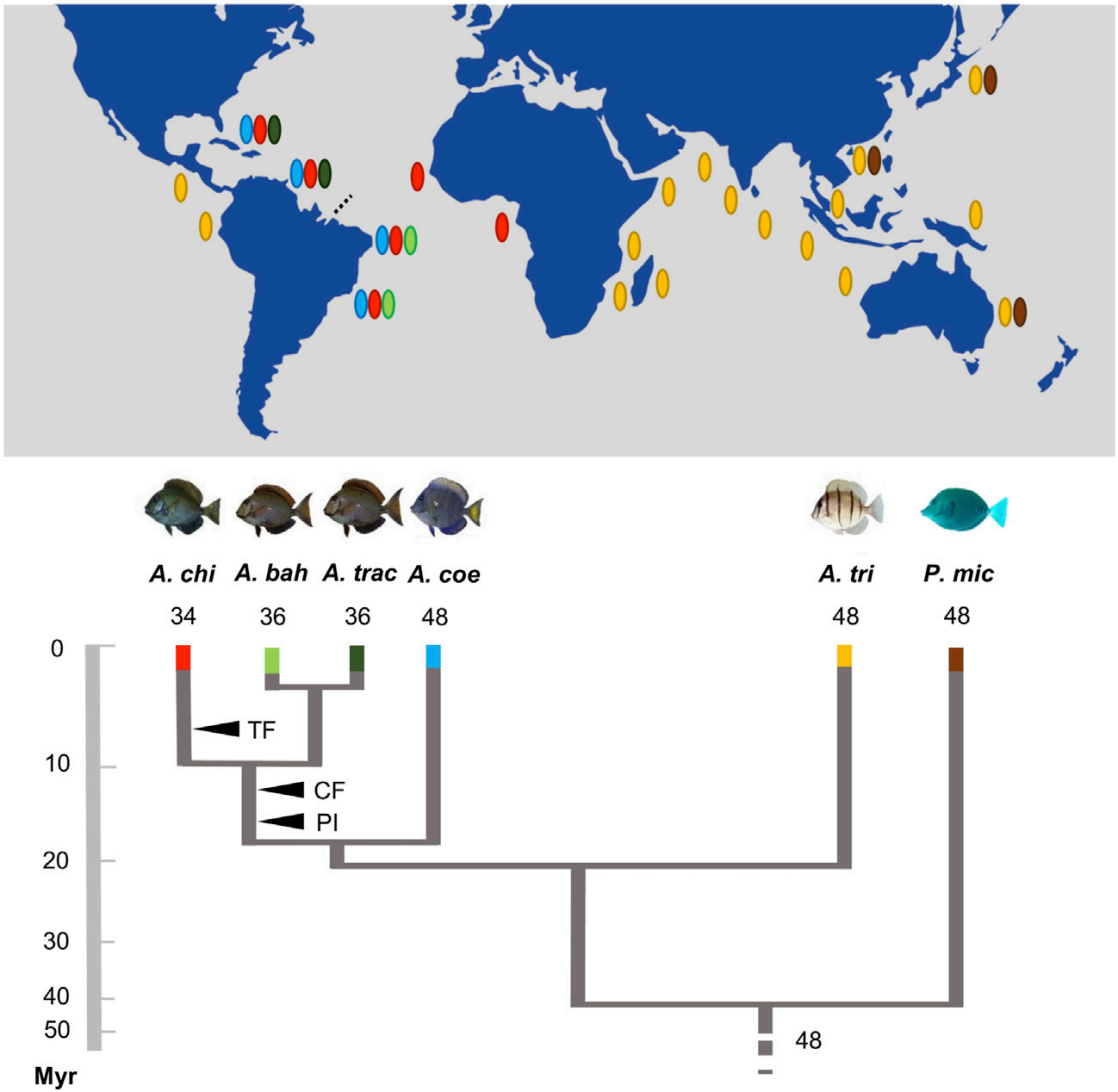
Karyotype diversification, phylogenetic relationships, and schematic geographic distribution of the *Acanthurus* species analyzed. The black arrowheads indicate shared and exclusive chromosomal rearrangements in the Atlantic species (CF: six centric fusions events; PI: three pericentric inversions events; TF: in tandem fusion) regarding their phylogenetic diversification (adapted from Bernal and Rocha, 2011; Sorenson et al., 2013). The dotted line highlights the Amazonas River plume. The names of the species are identified by abbreviations—*Acanthurus chirurgus (A. chi)*; *Acanthurus bahianus (A. bah)*; *Acanthurus tractus (A. tra)*; *Acanthurus coeruleus (A. coe)*; *Acanthurus triostegus (A. tri)*; *Prionurus microlepidotus (P. mic)*. It shows an inverted geographic distribution to the species *Acanthurus tractus* and *Acanthurus bahianus* analyzed.

The author apologizes for this error and state that this does not change the scientific conclusions of the article in any way. The original article has been updated.

